# Supplementary material for: Rapid assessment of avoidable blindness and cataract surgery coverage among forcibly displaced Myanmar Nationals (Rohingya refugees) in Cox’s Bazar, Bangladesh
Source: PLoS One. 2020 Dec 1;15(12):e0243005. doi: 10.1371/journal.pone.0243005 (PMC7707460; doi:10.1371/journal.pone.0243005)
Supplement: S1 File — (PDF) [file pone.0243005.s001.pdf]

S2. STROBE Checklist —Checklist of items that should be included in reports of ***cross-sectional studies***

	Item No	Recommendation	Page No
**Title and abstract**	1	(*a*) Indicate the study's design with a commonly used term in the title or the abstract	**Title Page page 1 – abstract**
		(*b*) Provide in the abstract an informative and balanced summary of what was done and what was found	**Pages 1-2 abstract**
**Introduction**			
Background/rationale	2	Explain the scientific background and rationale for the investigation being reported	**Pages 2-3 Introduction section**
Objectives	3	State specific objectives, including any prespecified hypotheses	**Page 3 Introduction section (lines 65-66)**
**Methods**			
Study design	4	Present key elements of study design early in the paper	**Page 3 Method. section Paragraph 1**
Setting	5	Describe the setting, locations, and relevant dates, including periods of recruitment, exposure, follow-up, and data collection	**Page 4 (study population) Page 5 (data collection)**
Participants	6	(*a*) Give the eligibility criteria, and the sources and methods of selection of participants	**Page 4 (study population) (sample selection)**
Variables	7	Clearly define all outcomes, exposures, predictors, potential confounders, and effect modifiers. Give diagnostic criteria, if applicable	**Page 5 (data collection) (ophthalmic exam) Page 6 (data management) Paragraph 2**
Data sources/ measurement	8*	For each variable of interest, give sources of data and details of methods of assessment (measurement). Describe comparability of assessment methods if there is more than one group	**Page 5 (ophthalmic exam) Page 6 (data management) Paragraph 2**
Bias	9	Describe any efforts to address potential sources of bias	**Page 5 (data collection) Page 6 (training & quality) Page 7-8 (data management)**

Study size	10	Explain how the study size was arrived at	**Page 4 (sample size)**
Quantitative variables	11	Explain how quantitative variables were handled in the analyses. If applicable, describe which groupings were chosen and why	**Page 7-8 (data management)**
Statistical methods	12	(*a*) Describe all statistical methods, including those used to control for confounding	**Page 7-8 (data management)**
		(*b*) Describe any methods used to examine subgroups and interactions	**Page 7-8 (data management)**
		(*c*) Explain how missing data were addressed	**N/A**
		(*d*) If applicable, describe analytical methods taking account of sampling strategy	**Page 7-8 (data management)**
		(*e*) Describe any sensitivity analyses	**N/A**
**Results**			
Participants	13*	(a) Report numbers of individuals at each stage of study—eg numbers potentially eligible, examined for eligibility, confirmed eligible, included in the study, completing follow-up, and analysed	**Page 4 (study population) Page 4 (sample size) Page 4 (sample selection) Page 8 (results) Page 9 Table 1**
		(b) Give reasons for non-participation at each stage	**Page 8 Results**
		(c) Consider use of a flow diagram	**N/A**
Descriptive data	14*	(a) Give characteristics of study participants (eg demographic, clinical, social) and information on exposures and potential confounders	**Page 9 Table 1**
		(b) Indicate number of participants with missing data for each variable of interest	**Page 8 Results**
Outcome data	15*	Report numbers of outcome events or summary measures	**Page 9 (Prev & cause BVI) Page 10 Table 2 Page 11 Table 3 Page 12 (cat coverage & vision outcomes) Page 13**

			**Table 4**<br>**Page 14**<br>**(Barriers to uptake)**<br>**(RE & presbyopia coverage)**<br>**Page 15**<br>**(extrapolation to population)**
Main results	16	(*a*) Give unadjusted estimates and, if applicable, confounder-adjusted estimates and their precision (eg, 95% confidence interval). Make clear which confounders were adjusted for and why they were included	**Page 9**<br>**(Prev & cause BVI)**<br>**Page 10**<br>**Table 2**
		(*b*) Report category boundaries when continuous variables were categorized	**N/A**
		(*c*) If relevant, consider translating estimates of relative risk into absolute risk for a meaningful time period	**N/A**
Other analyses	17	Report other analyses done—eg analyses of subgroups and interactions, and sensitivity analyses	**N/A**
**Discussion**			
Key results	18	Summarise key results with reference to study objectives	**Page 15**<br>**Discussion**<br>**Page 17**<br>**Paragraph 1**
Limitations	19	Discuss limitations of the study, taking into account sources of potential bias or imprecision. Discuss both direction and magnitude of any potential bias	**Page 17**<br>**(discussion)**<br>**Paragraph 2**
Interpretation	20	Give a cautious overall interpretation of results considering objectives, limitations, multiplicity of analyses, results from similar studies, and other relevant evidence	**Page 15-18**<br>**(discussion)**
Generalisability	21	Discuss the generalisability (external validity) of the study results	**Page 15**<br>**Discussion**<br>**Paragraph 1**<br>**Page 18**<br>**Last paragraph**
**Other information**			
Funding	22	Give the source of funding and the role of the funders for the present study and, if applicable, for the original study on which the present article is based	**Information provided**<br>**But not in manuscript**

*Give information separately for exposed and unexposed groups.

Note: An Explanation and Elaboration article discusses each checklist item and gives methodological background and published examples of transparent reporting. The STROBE checklist is best used in conjunction with this article (freely available on the Web sites of PLoS Medicine at http://www.plosmedicine.org/, Annals of Internal Medicine at http://www.annals.org/, and Epidemiology at http://www.epidem.com/). Information on the STROBE Initiative is available at www.strobe-statement.org.
